# Genomics of pediatric cardiomyopathy

**DOI:** 10.1038/s41390-025-03819-2

**Published:** 2025-02-08

**Authors:** Teresa M. Lee, Stephanie M. Ware, Alicia M. Kamsheh, Surbhi Bhatnagar, Mohammed Absi, Elyse Miller, Enkhsaikhan Purevjav, Kaitlin A. Ryan, Jeffrey A. Towbin, Steven E. Lipshultz

**Affiliations:** 1 Department of Pediatrics, Columbia University Medical Center, New York, NY USA; 2 Departments of Pediatrics and Medical and Molecular Genetics, Indiana University School of Medicine, Indianapolis, IN USA; 3 Department of Pediatrics, Washington University School of Medicine in St. Louis, St. Louis, MO USA; 4 Division of Biomedical Informatics, Cincinnati Children’s Hospital Medical Center, Cincinnati, OH USA; 5 Heart Institute, Division of Pediatric Cardiology, Le Bonheur Children’s Hospital, University of Tennessee Health Science Center, Memphis, TN USA; 6 Department of Pediatrics, University at Buffalo Jacobs School of Medicine and Biomedical Sciences, Clinical and Translational Research Center, Buffalo, NY USA

## Abstract

**Abstract:**

Cardiomyopathy in children is a leading cause of heart failure and cardiac transplantation. Disease-associated genetic variants play a significant role in the development of the different subtypes of disease. Genetic testing is increasingly being recognized as the standard of care for diagnosing this heterogeneous group of disorders, guiding management, providing prognostic information, and facilitating family-based risk stratification. The increase in clinical and research genetic testing within the field has led to new insights into this group of disorders. Mutations in genes encoding sarcomere, cytoskeletal, Z-disk, and sarcolemma proteins appear to play a major role in causing the overlapping clinical phenotypes called cardioskeletal myopathies through “final common pathway” links. For myocarditis, the high frequency of infectious exposures and wide spectrum of presentation suggest that genetic factors mediate the development and course of the disease, including genetic risk alleles, an association with cardiomyopathy, and undiagnosed arrhythmogenic cardiomyopathy. Finally, while we have made strides in elucidating the genetic architecture of pediatric cardiomyopathy, understanding the clinical implications of variants of uncertain significance remains a major issue. The need for continued genetic innovation in this field remains great, particularly as a basis to drive forward targeted precision medicine and gene therapy efforts.

**Impact:**

- Cardiomyopathy and skeletal myopathy can occur in the same patient secondary to gene mutations that encode for sarcomeric or cytoskeletal proteins, which are expressed in both muscle groups, highlighting that there are common final pathways of disease.
- The heterogeneous presentation of myocarditis is likely secondary to a complex interaction of multiple environmental and genetic factors, suggesting a utility to genetic testing in pediatric patients with myocarditis, particularly those in higher risk groups.
- Given the high prevalence of variants of uncertain significance in genetic testing, better bioinformatic tools and pipelines are needed to resolve their clinical meaning.

## Introduction

Cardiomyopathy in children is a leading cause of heart failure and cardiac transplantation globally. Disease-associated genetic variants play a significant role in the development of the different subtypes of pediatric cardiomyopathy. Over the past decades, enormous efforts have been made to collect clinical and outcome data using cohorts of children with cardiomyopathy and specific genotypes, advancing the study of cardiomyopathy genomics. Accurate determination of genetically associated cardiomyopathy helps develop targeted prevention strategies, particularly since pediatric cardiomyopathy frequently has a genetic basis.

Children with cardiomyopathy have been classified based on phenotypic characteristics that include cardiac imaging findings as well as the presence or absence of extracardiac features. Cardiac phenotypes include dilated cardiomyopathy (DCM), hypertrophic cardiomyopathy (HCM), restrictive cardiomyopathy, arrhythmogenic cardiomyopathy (ACM), and noncompaction cardiomyopathy. Pathogenic variants in genes encoding sarcomeric, cytoskeletal, or desmosomal proteins are frequent genetic causes of autosomal dominant cardiomyopathy in adults that can also present in childhood. However, syndromic, metabolic, and neuromuscular causes of cardiomyopathy are additional causes of cardiomyopathy in childhood. While some children have apparent extracardiac features that are pathognomonic for these conditions, other cases may be quite subtle and require a high degree of clinical suspicion. Guidelines recommend genetic testing in pediatric patients with cardiomyopathy. They are increasingly recognized as the standard of care for diagnosing heterogeneous genetic causes in the pediatric population, facilitating appropriate surveillance and management, providing prognostic information, and allowing family-based risk stratification.^1,2^ Gene-specific or disease-specific therapies are rapidly being developed, requiring providers to understand etiologic causes and tools for diagnosis. Understanding opportunities and limitations has become increasingly crucial as genetic testing approaches expand and develop technical sophistication.

This review provides a brief overview of pediatric cardiomyopathy with a specific focus on overlaps with skeletal myopathy and a discussion of new insights into the complex interplay between genetics and the environment in myocarditis. Our team’s research on the genetic architecture of pediatric cardiomyopathy is discussed along with the challenges of making inroads to understand more complex oligogenic or multigenic causality and how to build pipelines to resolve issues with variant interpretation. Finally, the current state of the art for clinical practice is reviewed and gene-specific therapies are highlighted along with the emergence of future treatments. We hope to offer pertinent background to bring the reader up to date on progress in the field while highlighting the current gaps in knowledge that must be addressed to propel the field forward.

## Primary cardiomyopathy

Frequent and often life-threatening symptoms of congestive heart failure (CHF), arrhythmias, sudden cardiac death (SCD), and the need for heart transplantation are hallmarks of all cardiomyopathy subtypes.^3^ The different cardiomyopathy types are characterized by changes in cardiac chamber size, thickness and stiffness of the myocardial walls, underlying cardiac function, and rhythm disturbances.^3^

DCM is characterized by left ventricular dilation and decreased systolic function; HCM is characterized by left ventricular hypertrophy with stiff ventricular function due to diastolic dysfunction; and restrictive cardiomyopathy is also accompanied by increased stiffness of the myocardium and a dilated left atrium due to diastolic dysfunction, but without significant hypertrophy.^4–6^ The main hallmark of ACM includes frequent and often life-threatening ventricular tachyarrhythmias and SCD.^7,8^

Noncompaction is a structural finding of the myocardium characterized by a spongy, noncompacted myocardium that appears as finger-like strips of myocardium with intertrabecular recesses separating a given trabeculation from another.^9–11^ Ventricular noncompaction was previously considered a distinct cardiomyopathy—though recently there has been controversy in regard to this, as it may be found in healthy controls—including physiologic states such as athletes and pregnancy, as well associated with other cardiac diseases such as congenital heart disease and other cardiomyopathy subtypes.^12–15^ Noncompaction, which has also been called left ventricular hypertrabeculation, may be more of a phenotypic trait rather than an isolated cardiomyopathy.

DCM is the most common form of primary cardiomyopathy that may present with signs and symptoms of heart failure or be asymptomatic^16,17^ and only identified on screening echocardiography.^18^ Newborns and infants tend to have dyspnea—especially with feeds—diaphoresis and failure to thrive; while young children and adolescents demonstrate exercise intolerance, dyspnea on exertion, easy fatigability with occasional chest pain, palpitations, syncope, and SCD.^5^ Ventricular tachyarrhythmias and conduction system disease may complicate the clinical features as well.^8,19^ Mutations in critical genes were previously identified in familial and sporadic cases of DCM by our group with age of clinical presentation starting in early childhood associated with relatively rapid progression to transplantation.^5,20^

## Dilated cardiomyopathy with overlapping phenotypes

Pediatric heart failure experts specialize in cardiomyopathy and heart failure care for children with all forms of heart muscle disease, while pediatric neuromuscular specialists care for children with all forms of skeletal myopathy, including muscular dystrophies and metabolic disorders such as Danon disease. While these clinical phenotypes may appear unrelated when compared by traditional clinicopathological criteria, recent evidence indicates they share common genetic causes and follow similar “final common pathways” as originally proposed by Towbin and colleagues.^21^ Mutations in genes encoding sarcomere, cytoskeletal, Z-disk, and sarcolemma proteins appear to play a major role in causing the overlapping clinical phenotypes called cardioskeletal myopathies in humans and animal models.^22^

Disease-causing variants are identified in only ~30–50% of patients and it appears that we lack knowledge regarding whether multiple mutations and/or modifying factors (e.g. effects of other gene variants, environmental factors, treatments) contribute to the progression of the disease.^23,24^ Thus, more work needs to be done to characterize cardioskeletal myopathies in view of genetic and “final common pathway” links.^21^ The development of a consistent and precise phenotyping strategy in patients presenting with primary cardiomyopathic features that will facilitate the identification of skeletal muscle health and vice versa is critically important. Furthermore, identification of the genetic basis of all forms of cardioskeletal disease using a comprehensive primary genotyping approach will allow clinically valuable correlation with outcomes and facilitate understanding of detailed mechanisms of phenotypes to improve risk stratification and to better predict adverse events. The increase in genotype-phenotype knowledge would greatly benefit prevention and personalized, targeted treatment strategies. For example, loss of healthy mitochondria is a pathological feature of both cardiac and skeletal myopathies, representing the common underlying mechanism that links directly with reduced energy supply, increased reactive oxygen species, and apoptosis resulting in progressive cardiac or skeletal muscle dysfunction and deterioration.^3,23–26^ Thus, restoring mitochondrial number and function would represent an effective way to treat cardiac and skeletal muscle diseases.^4,5^

## Skeletal muscle function in primary dilated cardiomyopathy

Children with left ventricular dysfunction due to primary cardiomyopathy complain of exercise intolerance and persistent fatigue during episodes of acute CHF as well as posttreatment. The processes by which exercise is limited in CHF remain unclear. There is poor correlation between exercise capacity and central hemodynamic measurements, and acute improvements in cardiac function do not result in an immediate improvement in exercise capacity.^27^ In addition, it has been well shown in adults that exercise intolerance is not determined directly by the extent of central hemodynamic disturbance.^28^ Yet, muscle bulk is known to be reduced in adults with chronic CHF, and 63–93% of patients experience tiredness with general muscle and fat tissue wasting predicting impaired survival in chronic disease.^29,30^ Also, abnormalities of skeletal muscle function, metabolism, and histologic features have all been described in adults with left ventricular dysfunction.^31,32^ It is possible that these muscle changes are part of the syndrome of chronic CHF and contribute to the symptoms of these patients,^33^ thereby suggesting muscle changes might be central to the exercise limitations seen in CHF and that these changes have important clinical implications.^34^

Furthermore, since respiratory muscle is also skeletal muscle, it is possible that the effects here also add to the clinical disorder.^35^ There is a strong relationship between skeletal muscle phenotype and maximal peak O_2_ consumption in healthy individuals, and the prognosis in CHF has been shown to depend on hemodynamic parameters and impaired functional capacity, particularly peak O_2_ consumption.^36–38^

## Cardiac function in primary skeletal myopathy

Primary skeletal myopathies with hypotonia and muscle weakness in the pediatric population also involve other organs or tissues and may result in significant morbidity and mortality.^26,39,40^ For example, limb-girdle muscular dystrophy is a group of disorders characterized by progressive muscle weakness affecting both upper and lower limbs. They mainly have autosomal recessive inheritance, elevated creatine kinase, and are due to mutations in sarcoglycans proteins.^41^ The electrocardiograms of these patients can have left ventricular hypertrophy and potentially late-onset DCM.^42,43^ One subtype is due to mutations in desmin (*DES*), the muscle-specific member of the intermediate filament which is localized to the periphery of the Z-lines where it links myofibrils to each other and to other intracellular structures including the sarcolemma, mitochondria, and the nucleus. To date, several mutations in desmin have been identified in patients with myopathy and/or cardiomyopathy.^44–46^

Another key cytoskeletal protein found to cause DCM is lamin A/C (*LMNA*), a nuclear envelope protein. Mutations in *LMNA* were initially identified as the cause of autosomal dominant Emery-Dreifuss muscular dystrophy with clinical features nearly identical to X-linked Emery-Dreifuss muscular dystrophy caused by emerin (*EMD*) mutations.^47^ Mutations in *LMNA* appear to be common in patients with DCM with an associated skeletal myopathy. Both *LMNA* and *EMD* result in conduction system disease initially with later onset DCM^48–51^ However, the mechanisms responsible for the development of conduction defects via disruption of nuclear lamina proteins are unclear.

Cardiac disease is now appearing to be common in most, if not all, skeletal myopathies, supporting the initial hypothesis that the cytoskeletal proteins play key roles in sarcolemmal structural support and, when mutated, lead to sarcolemmal disturbance and perturbation of the linkage of sarcolemma and sarcomere.

## “Muscle is muscle”

**Fig. 1:**
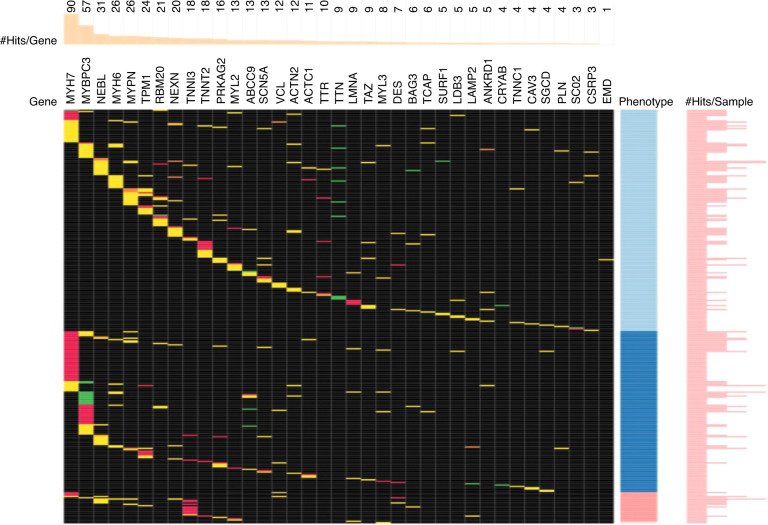
Cardiomyopathy genes expressed in both cardiac and skeletal muscle. Variant clustering by gene (x-axis) and phenotype (y-axis) with light blue–dilated (*n* = 279), dark blue–hypertrophic (*n* = 160), and pink–restrictive (*n* = 30) cardiomyopathy. Each row represents an individual participant, and each column represents a gene. Variants are color-coded as green–nonsense pathogenic, red–missense pathogenic, and yellow–missense of unknown significance.

To summarize, the clinical phenotype of DCM (left ventricular dilation and dysfunction), ventricular tachyarrhythmias, and conduction disturbances can occur due to triggered abnormalities of the sarcolemma-sarcomere linkage by the cytoskeletal mutation and a secondary cascade of events. These events result in a domino effect of contractile dysfunction (via sarcomere disturbance), dilation (via sarcolemmal disturbance), and rhythm abnormalities (via membrane disruption leading to the perturbance of membrane-bound ion channels and junction-junction contacts). Furthermore, we hypothesize that the extracardiac clinical features, particularly those that include easy fatigability and exercise intolerance with associated skeletal muscle deconditioning, muscle atrophy, and abnormalities of skeletal muscle oxidative metabolism result from primary expression in skeletal muscle of the mutant proteins encoded by the same cardiomyopathy-causing gene and its downstream effects on mitochondrial function. In other words, we suggest the concept that “muscle is muscle” and that patients with DCM have a genetic basis underlying skeletal myopathy which requires mechanical stress forces to develop clinically (Fig. 1).

As shown in Table 1, there are many cardiomyopathy genes that are also known to cause congenital skeletal myopathies and muscular dystrophies, and these pathologies have been diagnosed and managed separately due to their differing clinical manifestations. Despite progress in understanding the origin and progression of classic cardiac and skeletal myopathies,^25,26^ neither specialty team is equipped to identify dysfunction in the other muscle system, therefore, patients may go undiagnosed and untreated for extended periods, sometimes with disastrous outcomes such as sudden death or significant disability. There is a critical need to close the gap in the diagnosis and treatment of both types of muscle disease requiring collaboration between cardiomyopathy and skeletal myopathy experts to develop standardized diagnostic approaches with medical and gene therapies to alter the course of these maladies.

**Table 1:** Genes associated with common forms of cardiomyopathy with relation to skeletal muscle disease.

Genes	Cardiac phenotype	Syndromes/Skeletal myopathy phenotypes
*ABCC9*	DCM	Cantú syndrome Intellectual disability and myopathy syndrome
*ACTA1*	DCM^156,157^	Actin myopathy, nemaline myopathy, progressive scapulohumeroperoneal distal myopathy
*ACTC1*	DCM, HCM, NCM	Distal arthrogryposis
*ACTN2*	DCM, HCM, NCM	Distal myopathy, multiple structured core disease, myofibrillar myopathy
*ANKRD1*	ACM, DCM, HCM^158^	Congenital myasthenic syndrome, muscular dystrophy
*BAG3*	DCM	Limb-girdle muscular dystrophy, myofibrillar myopathy
*CAV3*	HCM	Distal myopathy, Emery-Dreifuss muscular dystrophy, limb-girdle muscular dystrophy, rippling muscle disease
*CRYAB*	DCM	Myofibrillar myopathy
*CSRP3*	DCM, HCM, NCM Endomyocardial fibroelastosis	Limb-girdle muscular dystrophy^159,160^
*DES*	ACM, DCM, NCM,^161^ RCM^162^	Myofibrillar myopathy, limb-girdle muscular dystrophy^159^
*DMD*	DCM	Duchenne muscular dystrophy, Becker muscular dystrophy
*EMD*	DCM	X-linked Emery-Dreifuss muscular dystrophy
*FLNC*	ACM, DCM,^163^ HCM, RCM	Distal myopathy, myofibrillar myopathy
*LAMP2*	DCM, HCM, NCM	Danon disease X-linked myopathy with excessive autophagy
*LDB3*	ACM, DCM	Myofibrillar myopathy
*LMNA*	ACM, DCM	Cap myopathy, Emery-Dreifuss muscular dystrophy, nemaline myopathy
*MYBPC3*	DCM, HCM, NCM	Myosin myopathy
*MYH7*	HCM, NCM	Congenital myopathy, distal myopathy
*MYL2*	DCM, HCM, RCM	Myofibrillar myopathy
*MYPN*	DCM, HCM, RCM	Cap myopathy, myopathy with hanging big toe, nemaline myopathy
*NEBL*	DCM, HCM Endomyocardial fibroelastosis	Limb-girdle muscular dystrophy
*SGCD*	DCM	Limb-girdle muscular dystrophy
*TAZ*	DCM, LVNC Endomyocardial fibroelastosis	Barth syndrome Myopathies
*TCAP*	ACM, DCM, HCM	Distal myopathy, limb-girdle muscular dystrophy
*TNNI3*	DCM, HCM, RCM	Distal arthrogryposis
*TNNT2*	DCM, HCM, RCM	Distal arthrogryposis
*TPM1*	DCM, HCM, LVNC	Nemaline myopathy
*TTN*	DCM, HCM	Hereditary myopathy with early respiratory failure, Salih myopathy, tardive tibial muscular dystrophy
*TTR*	Hereditary amyloid cardiomyopathy	Amyloid neuropathies

References provided for phenotypes not found in GeneCards or Online Inheritance of Man^156–163^.

*DCM* dilated cardiomyopathy, *HCM* hypertrophic cardiomyopathy, *NCM* noncompaction cardiomyopathy, *ACM* arrhythmogenic cardiomyopathy.

## Myocarditis

Myocarditis is a disease of myocardial inflammation with an estimated global incidence of 10–22 per 100,000 people.^52^ Studies suggest it is even more common in children, with an incidence in pediatric autopsy studies as high as 1.8%.^53^ Clinical phenotypes are heterogeneous and include subclinical disease, a mild coronary syndrome-like presentation, fulminant disease with heart failure and cardiogenic shock, and SCD. Most patients experience complete resolution of symptoms, others develop DCM and chronic heart failure, and still others require long-term mechanical circulatory support or heart transplantation.^53,54^ The underlying etiologies of myocarditis are also varied and include infectious, autoimmune, hypersensitivity, and toxic causes.^55^ Most frequently, acute myocarditis is associated with common viral infections.^56^ Some patients will clear the virus and improve while others will develop a chronic inflammatory state, which may be related to persistence of virus in the myocardium.^56–58^ Yet, the underlying explanation for why some patients develop myocarditis and what factors determine the clinical course of disease has not been fully elucidated.

## Genetic susceptibility

The commonness of the exposures as well as the diversity of presentations of acute myocarditis point to genetic factors which mediate the development and course of disease. Given this, multiple studies have been undertaken to better understand the genetic susceptibility to acute myocarditis. Initially, it was thought that genetic differences in the immunologic response likely accounted for differences in presentation. This idea is consistent with the fact that myocarditis can be seen in primary immunodeficiency, autoimmune, and autoinflammatory diseases.^59–63^ Major histocompatibility complex polymorphisms can predict autoimmune susceptibility and thus may relate to myocarditis predisposition. Several studies have examined groups of patients with idiopathic DCM, of which some cases may represent infectious or autoimmune myocarditis, and found an association with class II antigens, particularly HLA-DR4.^64–67^ However, these associations have not always been replicated in other studies.^66^

Given the importance of Toll-like receptors in the innate immune response to viral infection, Gorbea et al. studied 57 patients with enterovirus myocarditis and screened for variants in the *TLR3* gene. Within the myocarditis group, a patient carried a rare dominant-negative variant.^60^ As well, studies indicated that mice deficient in TLR3 and INF-β were more susceptible to Coxsackie virus infection and viral-induced heart injury.^68^

## Association with dilated cardiomyopathy

Belkaya and coauthors utilized whole exome sequencing to examine genetic variants in children with acute myocarditis.^69^ They found significant enrichment in biallelic—but not monoallelic—variants in cardiomyopathy-associated genes in patients with acute myocarditis as compared to controls. Approximately 17% of children with myocarditis carried these rare biallelic homozygous or compound heterozygote variants in cardiomyopathy genes.^69,70^ Pathogenic variants in these genes are typically associated with autosomal dominant genetic cardiomyopathy, but rare variants appeared to have an autosomal recessive mechanism in this cohort of patients with myocarditis.^69^

This data calls into question the historical notion that myocarditis and genetic cardiomyopathy are distinct entities. Clinical observations, including the fact that myocarditis is more common in certain genetic conditions with associated cardiomyopathy such as Duchenne muscular dystrophy, further dispute this concept.^71^ Multiple studies have since replicated the finding of an increased frequency of cardiomyopathy in patients with myocarditis.^72–76^ Artico et al. identified 11 out of 36 (31%) adult patients with biopsy-proven lymphocytic myocarditis to be variant carriers in structural cardiomyopathy-associated genes.^72^ Kontorovich et al. examined a cohort of predominately adults with acute myocarditis and found 19 of 117 (16%) patients harbored damaging variants in genes associated with cardiomyopathy or neuromuscular disorders, compared to only 7% of sex and ancestry-matched controls.^73^ These genes encoded for structural and functional proteins in the sarcolemma, desmosome, sarcomere, and other parts of the cytoskeleton. Lota et al. studied this association in 336 adult patients with myocarditis, which is the largest cohort of patients to date.^75^ A significantly higher rate of pathogenic variants in DCM or ACM-associated genes was again seen, though in a lower percentage of patients (8%). In this cohort, *DSP* mutations were most common in those with preserved ejection fraction, and *TTN* mutations were most common in those with reduced ejection fraction. While there have been fewer studies of this association in children, Kontorovich et al. did see a similar result in a small cohort of pediatric patients with death secondary to acute myocarditis.^74^ Monda et al. recently completed a systematic review and meta-analysis of studies examining the prevalence of cardiomyopathy-associated gene variants in patients with acute myocarditis.^77^ Authors calculated a pooled prevalence of 4.2% in uncomplicated myocarditis, 21.9% in adults with complicated myocarditis, and 44.5% in children with complicated myocarditis. Despite including all the studies examining the question to date, these pooled prevalences were based on relatively small groups of patients overall (n = 286, n = 209, and n = 47, respectively).

Importantly, there is data indicating that identification of a cardiomyopathy-associated pathogenic gene variant may be predictive of patient outcome. In the Lota et al. study, genetic variants were more frequently identified in patients with a lower ejection fraction and there was a trend toward greater all-cause mortality in this group.^75^ Another study of pediatric patients with biopsy-proven myocarditis found that patients who carried a heterozygous pathogenic or likely pathogenic variant were younger, more commonly had a DCM phenotype, and had worse freedom from mechanical circulatory support, heart transplantation, or death.^78^ However, other studies have not demonstrated a difference in outcomes between cardiomyopathy-associated gene-positive and gene-negative individuals.^73^

The underlying mechanism behind this association remains unclear. One possible explanation is a two-hit hypothesis, in which patients carrying cardiomyopathy-associated variants have a genetic predisposition to myocardial dysfunction with less reserve and thus have increased vulnerability to a second hit such as a viral infection.^73–75^ Thus, the environmental trigger unmasks the cardiomyopathy phenotype. This may be similar to other forms of acquired cardiomyopathies such as peripartum cardiomyopathy, chemotherapy-induced cardiomyopathy, and systemic immune-mediated disease-related cardiomyopathy, which have all been shown to have similar genetic predispositions as DCM.^79–81^ Alternatively, gene variants may decrease cardiomyocyte integrity, which may lead to increased susceptibility to seeding by a virus, aid in viral replication, or improve viral exit and propagation.^57,69,82–84^ There is evidence in mouse models that cardiomyopathy-associated proteins are directly involved in viral replication and release.^85^ Enterovirus viral proteases have been shown to cleave cardiomyocyte structural proteins dystrophin and dysferlin, and inducing cleavage-resistant dystrophin produces mice that are resistant to myocarditis.^83,85,86^ Furthermore, dystrophin-deficient mice infected with coxsackievirus have increased sarcolemma disruption, leading to increased viral release and more severe cardiomyopathy than wild-type mice.^82^

## Undiagnosed arrhythmogenic cardiomyopathy

Another reason for the association is that at least a portion of patients are misdiagnosed with myocarditis and that their inflammatory event is an early manifestation of ACM. New evidence suggests that inflammatory episodes may be part of an active phase of ACM and are not necessarily triggered by infection.^87,88^ These early active or “hot phases” of ACM closely mimic acute infectious myocarditis but are part of the natural history of this cardiomyopathy subtype.^75,76,89–92^ There are reported cases of myocarditis and recurrent myocarditis in patients with desmosomal gene mutations who have not yet developed overt pathologic findings of ACM.^84,92^ Identifying these patients is important, as patients with acute myocarditis and a desmosomal gene variant have been shown to be at higher risk for more severe outcomes, particularly myocarditis recurrence and arrhythmias.^89^

Data suggest that ACM is underrecognized in pediatrics due to the fact that it more commonly manifests as biventricular disease and presents with heart failure in children.^93^ Some pediatric cases of ACM are only diagnosed based on pathologic findings after heart transplantation and, therefore, ACM may be easily confused for infectious myocarditis. A case series of 16 patients with clinically diagnosed myocarditis with sustained ventricular arrhythmias or right ventricular abnormalities demonstrated a yield of 56% of pathogenic or likely pathogenic variants, most commonly in ACM-associated genes.^91^

**Fig. 2:**
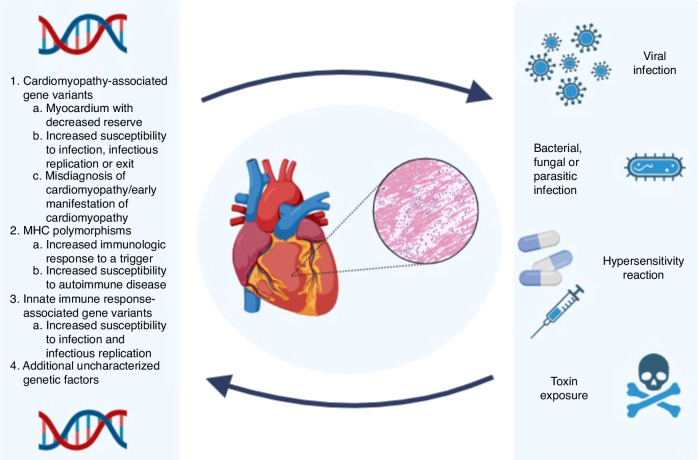
Complex gene and environmental factors interact to influence the development and clinical course of acute myocarditis. Created in BioRender. Kamsheh, A. (2024) https://BioRender.com/b95e843.

The heterogeneous presentation of myocarditis is likely secondary to the complex interaction of multiple of the aforementioned environmental and genetic factors, as well as additional yet uncharacterized gene variants which predispose to myocarditis (Fig. 2).^57,70^ We believe the data presented supports the consideration of genetic testing in pediatric patients with myocarditis, particularly those in higher risk groups including those with a dilated phenotype; low ejection fraction; ventricular arrhythmias; or a positive family history of myocarditis, cardiomyopathy, or sudden cardiac death.^75,77,89^

Additional studies, particularly larger and prospective studies, will be needed to better understand the gene and environment interactions determining which patients develop myocarditis and the expected clinical course. Larger studies of children are particularly imperative, given that, similar to cardiomyopathy, genetic variants leading to myocarditis in childhood are likely different than those leading to myocarditis in adulthood.^94^ The current standard of care in acute myocarditis remains predominately supportive. It is only with a better understanding of the mechanisms underlying the heterogenous course of the disease that we can improve outcome prediction, develop precision therapies, individualize follow-up care, and inform risk prediction and testing for family members.

## Genetic architecture of pediatric cardiomyopathy

Information on the genetic architecture of pediatric cardiomyopathy has lagged behind that of adults, partly because of the comparative rarity in children and the fact that genes and mechanisms causing cardiomyopathy in infants and children can differ from adults.^23,95–97^ While the cause of pediatric cardiomyopathy remains unknown in many cases, recent advances in genetic testing and sequencing technologies have provided new insights into the genetic architecture of pediatric cardiomyopathy, shedding light on the underlying mechanisms that contribute to the development of this condition.^94,98–105^ Next-generation DNA sequencing analysis has been adopted in routine diagnostics for genetic disorders and is a high-throughput discovery tool for identifying novel disease-causing genes. The availability of large public databases of genomic data has aided an understanding of causal genetic variation and has improved the stringency of variant interpretation.^106–111^

Studies have found pathogenic genetic variants responsible for up to 40% of all cases of pediatric cardiomyopathy, with abnormalities in genes encoding sarcomeric proteins being the most common cause.^112–114^ Sarcomeres are the basic units of muscle contraction and relaxation in the heart, and pathogenic variants in genes that encode these proteins can lead to structural abnormalities, impaired cardiac function, and, ultimately, heart failure. Other genetic variants associated with pediatric cardiomyopathy may impact ion channels, mitochondrial function, and structural cytoskeletal or desmosomal proteins.^115,116^ Abnormalities in these genes can disrupt the heart’s electrical and metabolic processes, leading to abnormal heart rhythms and impaired energy production.

In the investigation by Vasilescu et al., a country-wide cohort from Finland was used to investigate underlying genetic causes of severe early-onset cardiomyopathy over an 11-year period.^104^ All children aged 15 or less who required inotropic support or underwent transplant evaluation were included. While the total cohort was modest (*n* = 66), the study expanded the genes associated with cardiomyopathy by identifying causative variants in genes previously associated with arrhythmia (*CALM1*), genes associated with heart development, and genes (*PPA2*, *NRAP*) that may be susceptibility factors for cardiomyopathy in combination with environmental triggers such as viral infection. This study also highlighted the need for routine genetic evaluation because it frequently identified de novo variants and autosomal recessive disease, which has significantly different implications for family-based surveillance compared to sarcomeric autosomal dominant causes.

## Differences based on cardiomyopathy subtype

In our study on the genetic architecture of pediatric cardiomyopathy, the Pediatric Cardiomyopathy Genes study, we focused on children without apparent extracardiac or syndromic features and performed research-based exome sequencing on 528 children.^94,105^ Across all cardiomyopathy phenotypes, 32% had a pathogenic or likely pathogenic variant identified when analyzed for 37 genes clinically associated with cardiomyopathy. Although 53% of patients had already undergone clinical genetic testing, overall diagnoses were increased by 20% after testing all patients. This is a minimum estimate given that only 37 genes were interpreted using stringent clinical standards. The diagnostic yield was highest for HCM and RCM at 51% and 50%, respectively.

## Differences based on ancestry

When using bioinformatic predictions in Pediatric Cardiomyopathy Genes study participants compared to control cases, the damaging rare variant burden was significantly higher in cases. Overall, individuals of European descent with HCM were 4.38-fold more likely to have a rare damaging variant than controls, and for DCM, 2.83-fold more likely. Admixed American participants also had enriched damaging rare variant burden over controls, but African American participants did not. In addition, a lower burden of known pathogenic and likely pathogenic variants among known cardiomyopathy genes has been observed among African and African American ancestries. This may indicate alternative mechanisms and genes that may not be represented in cohorts primarily of people with European ancestry. Recent studies examining African or African American populations found additional genes of interest that may explain causal mechanisms among these groups.^117–121^ Further research is necessary to understand the differences in ancestry and the genetics of pediatric cardiomyopathy.

## Other genetic models of disease

Clinically, the focus of diagnostic genetic testing is on disease-causing variants that have high penetrance. As noted, this diagnostic evaluation is essential for distinguishing underlying causes that increasingly direct management and for risk stratification of family members. A portion of genetic disease in the pediatric cardiomyopathy population is likely oligogenic or multigenic, in which an individual carries genetic susceptibility due to combinations of rare or common variants in multiple genes impacting heart muscle function. Developing additional methods for identifying susceptibility alleles will be necessary to better understand disease pathogenesis. Phenotypic predictors based on molecular modeling and machine learning approaches are essential to develop and expand our understanding of multigenic and gene-environment multifactorial causation in pediatric cardiomyopathy.^122^

Finally, it is important to note that even in pediatric cardiomyopathy patients where an inherited pathogenic variant is identified, their presentation and disease course may be different from their family. The shared causes of cardiomyopathy in children and adults were first reported in 2008.^123^ While interfamilial variability in onset and severity is well documented, it is not well understood. Smaller studies and case reports have identified multiple pathogenic variants in well-characterized cardiomyopathy genes in children, but in the largest study to date, multiple variants in these genes did not meet statistical significance.^95,96^ It is likely that susceptibility alleles in genes remaining to be identified play an important role in modifying the onset of disease. This hypothesized gene-gene interaction is similar in principle to oligogenic inheritance or gene-environment interactions contributing to age of onset and presentation.

## Variant classification

With the development of next-generation sequencing technologies, the entire human genetic blueprint of 20,000 genes can be sequenced in a single run. However, as more individuals have been sequenced, the spectrum of normal genetic variation has dramatically increased, with the average person carrying about 24,000 variants in the protein-coding region alone.^124^ The current gold standard scoring system for variant interpretation is the American College of Medical Genetics and Genomics and the Association for Molecular Pathology guidelines, which categorizes variants into 5 groups: pathogenic, likely pathogenic, variant of uncertain significance (VUS), likely benign, and benign.^125^ Under these guidelines, only pathogenic and likely pathogenic variants can be used for predictive testing and medical decision-making. While some VUSs may be possibly pathogenic, without sufficient supporting evidence, VUSs cannot be used in patient management.

The 2015 standards and guidelines for the interpretation of sequence variants provided updated recommendations on features that should be taken into account in variant adjudication with a goal of developing more consensus between clinical genetic testing laboratories.^126^ As a result of these recommendations, computational predictions of variant effect which had previously been heavily relied upon for variant classification, became a feature that could provide supporting evidence of pathogenicity. These in silico tools for variant prediction are described in more detail in the next section.

Functional studies to support pathogenicity are additional features that can provide strong, moderate, or supporting evidence for pathogenicity depending on the type of functional evidence, gene, and mechanism of disease. In general, RNA studies demonstrating splicing abnormalities with protein disruption of nonsense-mediated decay are considered strong evidence of pathogenicity. ClinGen has recently provided detailed recommendations for the interpretation of potential splice variants. In vivo functional data such as mouse models are often considered moderate indicators of pathogenicity. Finally, in vitro functional data often provides supporting evidence of pathogenicity. Functional genomic studies can be time-consuming and costly and there are ongoing efforts in the field to develop higher throughput methodologies for functional genomic testing.^127^

Minor allele frequency from population databases continues to have an important place in variant classification and population control studies are becoming more important. In particular, it remains important to diversify public databases of genetic variants to ensure broad representation across ancestries. Variant classification is particularly challenging for patients from non-White populations, including Hispanic individuals, due to limited reference population data.^128^

Moreover, given the marked genetic heterogeneity associated with pediatric cardiomyopathy, many researchers and clinicians are turning to exome and/or genome sequencing.^129^ Copy number variants, small (>1000 base pair) deletions and duplications in the genome, are an important cause of many rare genetic diseases—especially in the pediatric population—but have been less studied with regard to their contribution to pediatric cardiomyopathy. It is likely that deletions of genes or promoters/enhancers of genes leading to loss of function will explain a proportion of cardiomyopathy cases that are not detected by clinical panel testing. Exome testing can identify gene deletions, although this is technically more challenging than with genome testing. Genome testing has the advantage of also being able to detect deletions of promoter or enhancer regions.

## Variants of uncertain significance

Genetic analyses are predominantly focused on pathogenic variants with almost no studies that examine the role that VUSs play in disease pathogenicity. This gap is surprising as almost a quarter of genomic sequencing results in infants with cardiac abnormalities are inconclusive with multiple uninterpretable VUSs.^129^ In our own targeted exome analysis on 528 children in the Pediatric Cardiomyopathy Genes study, 32% had a positive result (pathogenic or likely pathogenic) while 35% had a VUS result.^129^ We also found that 18% of individuals who had prior genetic testing had a change in variant classification with 82% of reclassifications resulting in a variant downgrade. Most of these were changes from the pathogenic or likely pathogenic category to a VUS.

Furthermore, many studies have suggested that VUSs may also act as important disease modifiers that influence heart failure severity and outcomes. While the concept of oligogenic inheritance—a few genes acting together to cause disease—is often mentioned, it has rarely been studied in the cardiomyopathy field. Research has established that the increased genetic burden of both pathogenic variants and VUSs is associated with worse clinical outcomes in pediatric cardiomyopathy, but we are yet to identify the true clinical significance of VUSs for pediatric cardiomyopathy despite their prevalence.^129^

## In silico tools

In recent years, there has been growing interest in using in silico predictions to help resolve VUSs. In silico predictions involve using computational models, learned on variants with known significance, to predict the functional effect of a genetic variant on protein structure and function to determine whether a VUS is likely to be damaging (disease-causing) or benign (not disease-causing). Recent advances in machine learning algorithms have improved the accuracy of predicting potentially damaging variants in known cardiomyopathy genes.^130–133^

Many different in silico prediction tools are available, each using a different algorithm and set of criteria to make predictions. Commonly used tools, such as SIFT, PolyPhen-2, and CADD, are used to obtain a consensus score for classifying and interpreting variants using the 2015 American College of Medical Genetics guidelines.^126,134–136^ Some studies have used these tools and recommended guidelines to resolve missense and loss-of-function variants in cardiomyopathy cohorts.^137–146^ Other popular tools used include MetaSVM and REVEL.^147,148^ Notably, the ClinGen variant interpretation framework utilizes REVEL scores to improve predictions for cardiomyopathy genes for HCM and DCM.^132,149^ The latest advances have inspired deep learning artificial intelligence-based tools such as AlphaMissense and PrimateAI to predict variant pathogenicity.^150^ They have been shown to have improved overall pathogenicity classification with greater coverage across variants.^151^

However, while in silico predictions can help resolve VUSs, they are not infallible. Most genome-wide prediction tools are reported to have low specificity.^126^ Many factors can influence the accuracy of in silico predictions, including the quality of the input data, the specific algorithms and criteria used, and the level of validation and testing performed on the prediction tool. Comparative studies have found that in silico prediction models are not one-size-fits-all, and their performance may vary. The best prediction tool may depend on the condition, and disease-specific variant pathogenicity prediction may significantly improve variant interpretation in cardiomyopathy.^94,144^ While some models work substantially better for particular conditions, these gains may be lost while implementing a consensus-based approach. Notably, a dedicated evaluation of cardiomyopathy has found MetaSVM, FATHMM, and MetaLR had better performance.^94,143^

## Stem cell modeling

Induced pluripotent stem cell-derived cardiomyocytes (iPSC-CMs) and cardiac bodies (organoids) have become powerful tools for modeling cardiomyopathy. CMs generated from patients with genetic forms of cardiomyopathy can be used to recapitulate key features of diseases like DCM. Since these cells maintain the genetic background of the patient, they can be particularly beneficial for understanding how specific genetic variants affect cardiac function at the cellular level. Additionally, 3D cardiac bodies—constructed from iPSC-CMs along with other cell types, such as endothelial and fibroblast cells—offer a more physiologically relevant environment that mimics heart tissue architecture and function. These models allow for the investigation of disease mechanisms such as contractile dysfunction and arrhythmias to provide a closer approximation of the in vivo disease state compared to traditional 2D cultures.^152^ Recent advances in the maturation of iPSC-CMs and cardiac bodies have made it possible to model complex aspects of the disease, including electrophysiological abnormalities and altered calcium handling seen in cardiomyopathy.^153,154^

Moreover, iPSC-CMs generated from individuals with various cardiomyopathy subtypes allow for the identification of novel biomarkers and the testing of therapeutics in a more relevant context. iPSC-derived models remain a promising approach for understanding cardiomyopathy at the cellular and molecular level, facilitating the development of novel therapeutic strategies and precision medicine approaches.

## Extending candidate gene sets using systems biology

Additional approaches can also be used to validate any VUS after in silico predictions using functional studies, such as in vitro and in vivo assays, which can directly measure the effect of a variant on protein function. These approaches can provide more direct and reliable information about the functional impact of a variant, but they are often more time-consuming and resource-intensive. Since in silico predictions are quick, expanding beyond the scope of known genes and evaluating disease-causing variants in novel genes is feasible. Systems biology approaches can be used to identify a candidate set of genes using known information about underlying mechanisms of action and pathways involved in the disease.

**Fig. 3:**
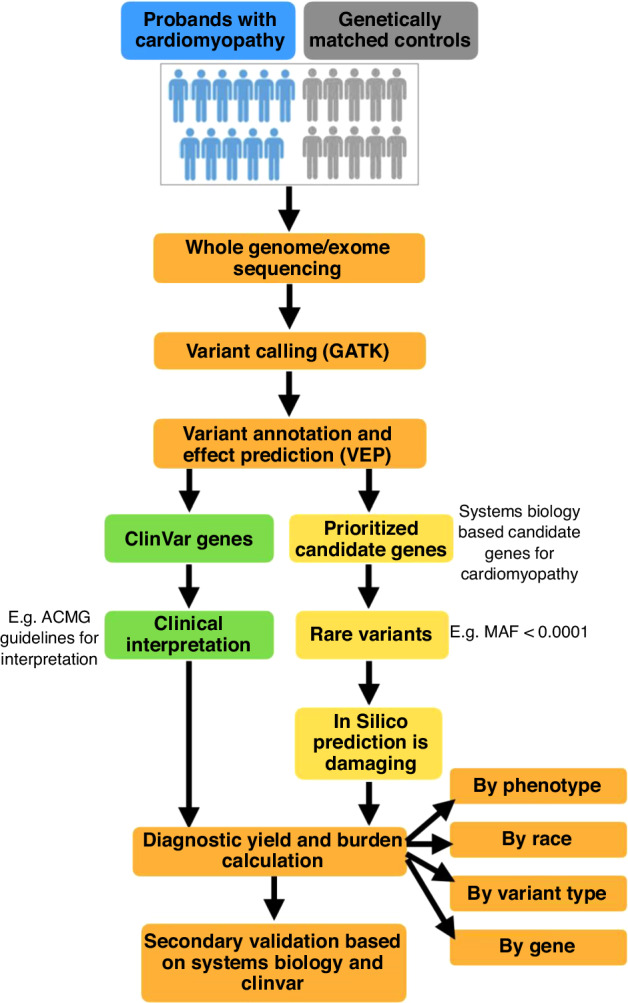
Overall workflow for a cardiomyopathy cohort’s genetic examination and interpretation.

For the Pediatric Cardiomyopathy Genes study, we found candidate genes for cardiomyopathy by compiling a list of genes known or associated with cardiomyopathy, heart development, and cardiac muscle structure using available human phenotype, mouse phenotype, and co-expression data.^94^ These candidate genes were further prioritized by identifying loss-of-function and missense-intolerant genes. In addition to these candidate genes, genes from ClinVar with annotations for cardiomyopathy were included. A workflow was developed in which bioinformatic in silico predictions and minor allele frequency were used to identify rare, potentially damaging alleles in both cases and controls (Fig. 3). The overall individual-level damaging rare variant burden was then compared in cases versus controls to investigate the hypothesis that rare predicted damaging variants in the larger candidate gene set would be overrepresented in cardiomyopathy cases versus controls. We further stratified the cohorts to look specifically at rare variant burden by phenotype (HCM, DCM etc.), ancestry (race/ethnicity), variant type, and gene. We predicted that this expanded gene set found potentially damaging variants for cardiomyopathy in novel genes and increased the diagnostic yield by 33%.

Many researchers studying specific genes need guidance to prioritize VUSs for functional validation. In particular, nonsynonymous single nucleotide variants exhibit a wide range of functional consequences, from benign to protein damaging. This high level of variability makes the classification of these variants’ pathogenicity particularly challenging. Since functional validation of these variants can be time-consuming and costly, computational methods have been developed. Given the large number of VUSs encountered in pediatric cardiomyopathy and the critical importance of having an accurate genetic diagnosis to initiate appropriate and lifesaving therapies, there is a need for better pipelines to resolve VUSs in clinical medicine.

## Novel treatments

There are many novel genetic therapeutic approaches to treating cardiomyopathy being validated. A recent example is the HELIOS-B clinical trial which demonstrated that a novel RNA interference therapeutic, vutrisiran, that targets transthyretin production, improved clinical outcomes in patients with cardiomyopathy. This is an example where this novel gene silencer therapy worked by binding to and stifling messenger RNA to reduce the disease-causing protein called transthyretin. Vutrisiran reduced the risk of death and recurring cardiovascular events_—_such as heart attacks or strokes—by 28% in patients with transthyretin amyloid cardiomyopathy.^155^

Other examples of gene-related therapies currently being investigated for cardiomyopathy include adeno-associated virus gene therapy to produce micro-dystrophin. Theoretically, adeno-associated virus gene therapy could be used to treat all forms of Duchenne muscular dystrophy, regardless of mutation, and would require only a single administration. If successful, micro-dystrophins would be among the first gene therapy for a form of childhood-onset cardiomyopathy.

Another example of a gene-related therapy for pediatric cardiomyopathy is that of precision gene editing techniques. Delivery of CRISPR/Cas9 nucleases capable of genome editing with delivery via adeno-associated virus vectors has been shown to be feasible in preclinical studies. These studies aim to restore the gene reading frame to produce a truncated but partially active protein. As in gene therapy, gene editing may require only a single administration. Again, given the affinity of certain adeno-associated virus serotypes for the heart, cardiac correction is expected.

## Conclusion

Substantial progress has been made in diagnostically identifying new genetic factors that have contributed to a deeper understanding of cardiomyopathy in children. The need for continued genetic innovation in this field remains great not only for providing the basis for targeted genetic-directed or genetic-specific therapies and clinical care but also for enhancing our understanding of the associated genetic variants in children for genetic counseling to improve personalized risk stratification and longitudinal screening as pathogenic variants may manifest in a time-dependent manner.

When cardiomyopathy is identified in a child without a known pre-existing risk, panel genetic testing or whole exome sequencing is recommended. A pathogenic or likely pathogenic variant in the proband should prompt cascade genetic testing of first-degree relatives who are at risk for cardiomyopathy. Cardiac surveillance is no longer necessary for family members with informative negative genetic test results. Both clinical surveillance and cascade genetic testing for first-degree relatives of probands with cardiomyopathy are important. Indeed, screening guidelines recommend a three-generation pedigree, cardiac screening, and cascade genetic testing for at-risk family members.

Our goals are to emphasize the significance of identifying genetic cardiomyopathy, the relationships between genotype and phenotype, risk assessment, and personalized therapy for those affected and their relatives. Understanding the molecular mechanisms underlying the development of pediatric cardiomyopathy and then the genotype-phenotype correlation and their implications for prognosis and treatment is important.

Integrated multi-modality registries, including joint biorepositories with genomic and clinical datasets, could be leveraged to speed the translation of research findings into clinical practice guidelines. Given the challenges of conducting randomized trials in children with cardiomyopathy, a collaborative platform is particularly suited to this field of research.
